# A Decoupled Unified Observation Method of Stochastic Multidimensional Vibration for Wind Tunnel Models

**DOI:** 10.3390/s20174694

**Published:** 2020-08-20

**Authors:** Mengde Zhou, Wei Liu, Qinqin Wang, Bing Liang, Linlin Tang, Yang Zhang, Xiaochun Cui

**Affiliations:** 1 Key Laboratory for Precision and Non-traditional Machining Technology of the Ministry of Education, Dalian University of Technology, Dalian 116024, China; mengde@mail.dlut.edu.cn (M.Z.); 305210715@dlut.edu.cn (Q.W.); liangbing2016@mail.dlut.edu.cn (B.L.); Tanglinlin1995@mail.dlut.edu.cn (L.T.); zy2018@dlut.edu.cn (Y.Z.); 2 AVIC Aerodynamics Research Institute, Shenyang 110034, China; cxiaochun@163.com

**Keywords:** wind tunnel, stochastic multidimensional vibration, decoupled observation, unified observation, accelerometer

## Abstract

Active vibration control is the most effective method for stochastic multidimensional vibration in wind tunnel tests, in which vibration monitoring is the core foundation. Vibrations are induced by the disturbances of several complex air flow instabilities under extreme test conditions with high attack angles. Here, a decoupled unified observation method is proposed in order to fully monitor stochastic multidimensional vibration. First, stochastic multidimensional vibration is explained using the Cartesian coordinate system. Then, the multidimensional vibration decoupling of the pitch plane and the yaw plane is realized according to the proposed decoupling design principle of the long cantilever sting. A unified observation method is presented, based on inertial force theory, to observe multidimensional vibration due to acceleration in each decoupling plane. Verification experiments were conducted in lab and a transonic wind tunnel, using an established real-time monitoring system. The results of lab experiments indicate that, in the frequency region of 0–120 Hz, three vibration modes of a selected stochastic vibration can be decoupled and observed through the vibration components in pitch plane and yaw plane. In addition, wind tunnel tests were carried out according to the working conditions (α = −4~10° with γ = 45°) at Ma = 0.6 and Ma = 0.7, respectively. The results show that six vibration modes of two selected stochastic vibrations can be decoupled and observed through the vibration components in pitch plane and yaw plane. The experimental results prove that stochastic vibration can be fully monitored in multiple dimensions through the vibration components in pitch plane and yaw plane using the proposed decoupled unified observation method. Therefore, these results lay the foundation for active vibration control.

## 1. Introduction

High-speed aircraft, such as large passenger airplanes and military aircraft, are high-end pieces of equipment in the aeronautical and space fields. In order to ensure performance, it is necessary to evaluate the aerodynamic characteristic of these aircraft at a range of speed and altitude. To evaluate the aerodynamic characteristic accurately, key aerodynamic data should be obtained under the conditions of a simulated flow field. Currently, the most effective and direct method is the wind tunnel test. During data acquisition tests for an aircraft’s overall performance, the aircraft model is mounted by the long cantilever tail-support [1]. Under extreme test conditions with high attack angles, the aircraft model is subject to disturbances of complex unstable airflow [2,3], thereby inducing the aircraft model to undergo serious stochastic multidimensional vibration [4]. These aerodynamic characteristics cannot be tested at all if the vibration is not effectively suppressed. At present, the most effective method to suppress this serious stochastic multidimensional vibration is the active vibration control method [5,6], wherein vibration is monitored as feedback by vibration sensors; after real-time calculations with a control strategy, actuators are driven to output reverse force or moment to suppress system vibration [7]. Vibration monitoring is the core foundation of active vibration control. Therefore, monitoring stochastic multidimensional vibration for multidimensional vibration active control is of great significance.

Two main methods are reported to monitor multidimensional vibration of aircraft models, namely, force or moment monitoring feedback and acceleration monitoring feedback. Hefer et al. [7,8,9] first gave an explanation of multidimensional vibration in their related research regarding active vibration control: The reciprocating fluctuation of six-dimensional aerodynamic load relative to stable values in the Cartesian coordinate system is the multidimensional vibration of the aircraft model. On this basis, Balakrishna et al. [7,8,9,10] monitored fluctuating multidimensional aerodynamic loads of aircraft models with aerodynamic load measuring sensors, with the axial force (AF), lateral force (SF), and normal force (NF) being intercepted as the vibration mechanical variable to monitor the three-dimensional vibration of the aircraft model in ViGYAN’s low-speed wind tunnel. The analysis of the vibration characteristics showed that one vibration mode was observed in the vibration of NF, and two vibration modes were observed in the vibration of SF.

Balakrishna [11] and Rivers [12] et al. further extended their dimension-reduction observations to monitor the multidimensional vibration of aircraft models in NASA’s Langley Research Center National Transitional Facility (NTF) and Ames Research Center 11 × 11 ft. The pitch moment (PM) and the lateral moment (YM) were selected to be the vibration mechanical variables used to monitor the multidimensional vibration of the aircraft models. For the serious stochastic multidimensional vibration of Pathfinder I at an attack angle of around 5°, one vibration mode and two vibration modes were respectively observed in the PM and YM vibrations. For the continuous unstable vibrations of 1% and 0.548% Crew Launch Vehicle (CLV), one vibration mode was observed for each PM and YM vibration.

Aceson et al. [13,14] employed the intercepted pitch moment YM and yaw moment PM as the vibration mechanical variables, which were used in a sting damper design for the Common Research Model (CRM) at the NTF from the viewpoint of vibration energy; the forced response damping enhancement was between 4 and 8 dB. Shen et al. [15,16,17] selected the original voltage signal of a force or moment of six-dimensional aerodynamic load to monitor the pitch vibration of the aircraft model; two vibration modes were observed in the pitch plane. The maximum attenuation of vibration reached 91% standard deviation, and the extension of the attack angle was 6°.

With the in-depth application of piezoelectric materials, the small, lightweight Integral Electronic Piezoelectric (IEPE) accelerometer was rapidly and widely applied in vibration monitoring of the aircraft model [18,19,20]. Ma et al. [21] focused on the vibration of aircraft model in pitch plane, setting three accelerometers on the cantilever support to analyze the characteristic of pitch transverse vibration, and observe two vibration modes. With active vibration control, the acceleration amplitude of the first mode reduced to 7.06%, and the second mode to 31.35%. Julien Weiss [22] used a triaxial accelerometer to measure the normal, side, and axial acceleration of the vibration for aircraft models in the North American Trisonic Wind Tunnel. The analysis showed that fluctuations in the aerodynamic load were due to the inertial forces on the aircraft model, which were caused by the unstable wide-band airflow disturbance. The qualitative analysis showed that the occurrence of serious rotating oscillation was impossible.

As mentioned above, only some of the vibration characteristics can be observed by intercepting single- or multidimensional force/moment in the active vibration control of the wind tunnel model. This leads to an unsatisfactory vibration control effect. In this paper, a decoupled unified observation method is proposed to lay the foundations of active vibration control. This method aims to solve the severe multidimensional vibration problem of the wind tunnel model, and fully monitor serious multidimensional vibration.

The rest of this paper is organized as follows. Section 2 introduces the stochastic multidimensional vibration according to the Cartesian coordinate system. Section 3 presents the analysis of the natural vibration characteristics of the cantilever support system, and proposes a decoupling design method of the cantilever sting. In Section 4, a unified method for multidimensional vibration observation is proposed. Based on the theory of inertial force, multidimensional vibration is observed by acceleration in the decoupling plane. Section 5 discusses the establishment of a real-time monitoring system and the performance of verification experiments. Section 6 summarizes this paper.

## 2. Outline of the Aircraft Model’s Multidimensional Vibration

**Figure 1:**
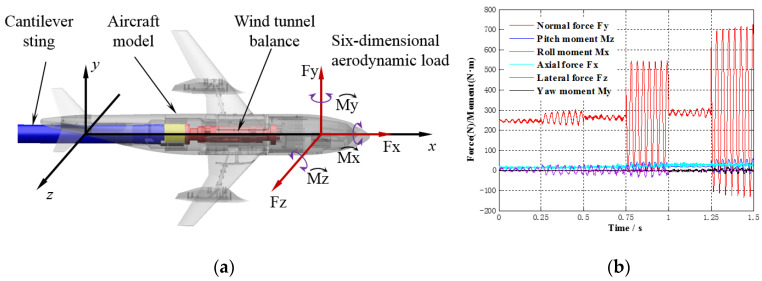
Stochastic multidimensional vibration of an aircraft model. (**a**) Definition of six-dimensional aerodynamic load. (**b**) Multidimensional vibration of an aircraft model.

In the wind tunnel test, an aircraft model is fixed by a long cantilever support composed of wind tunnel balance and the cantilever sting. The six-dimensional aerodynamic load in the Cartesian coordinate system is shown in Figure 1a, where the axis of the aircraft model is defined as the *x*-axis, the direction points to the nose of aircraft model, and the aerodynamic load component on the *x*-axis is the axial force Fx. The vertical axis in the pitch plane of the aircraft model is defined as the *y*-axis, and the aerodynamic load component on the *y*-axis is the normal force Fy. The *z*-axis is determined according to the right-hand rule, and the aerodynamic load component on the *z*-axis is the lateral force Fz. The moment components of the aerodynamic load on each axis are the roll moment Mx, the yaw moment My, and the pitch moment Mz. Under extreme test conditions with high attack angles, the disturbances in complex unstable airflow lead to large-amplitude reciprocating fluctuation relative to the stable force or moment, resulting in poor quality aerodynamic load data, or aerodynamic load sensor overload. As shown in Figure 1b, vibration mainly occurs in five of the six dimensions, excluding the rolling dimension [4]. To provide feedback for active vibration control, this paper conducts full-dimensional monitoring for serious multidimensional vibration.

## 3. Decoupling Design Principle of the Cantilever Sting

For the multidimensional vibration of an aircraft model, in order to complete the full-dimensional monitoring with the fewest observations, the natural vibration characteristics of the cantilever support system are qualitatively analyzed, according to Hamilton’s principle. Based on this, a decoupling design principle of a long cantilever sting is proposed, providing a structural basis for decoupling monitoring.

### 3.1. Vibration Characteristics Analysis Based on Hamilton’s Principle

The aerodynamic dynamic load of the long cantilever structure, which is composed of the aircraft model, wind tunnel balance, and the cantilever strut, is stochastic and unpredictable under the disturbance of various complicated unsteady airflow. This phenomenon leads to uncertainty of the relationship between the direction of the force and the plane of the principal inertia axis. With the excitation of the stochastic dynamic force, the vibration of the aircraft model supported by the long cantilever structure is beyond the research scope of classical transverse vibration theory. Hamilton’s principle is a basic variational principle, and is the most effective method to establish dynamic equations for large-scale structural systems with multiple degrees of freedom.

**Figure 2:**
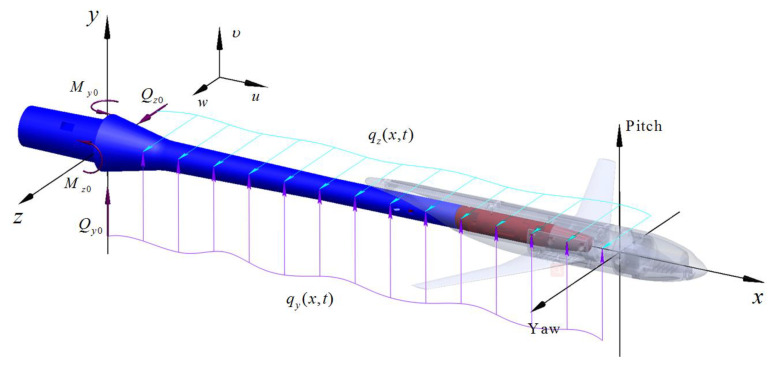
Long cantilever support system simplified into a Bernoulli beam.

As shown in Figure 2, the long cantilever system, composed of an aircraft model, wind tunnel balance, and the cantilever sting, is simplified as a Bernoulli beam, according to Euler Bernoulli beam theory. Then, the vibration characteristics are analyzed using Hamilton’s principle. Based on force decomposition, the external stochastic aerodynamic load is respectively simplified into distributed load. The distributed load $q_{y}(x,t)$ is distributed on the *y*-axis, and the distributed load $q_{z}(x,t)$ is distributed on the *z*-axis. The concentrated forces of the fixed support end are defined as $Q_{y0}$ and $Q_{z0}$, and the concentrated couples are $M_{y0}$ and $M_{z0}$. Generally, the cross-section’s main direction of the long cantilever structure changes with the axial direction, and the axis of the long cantilever structure no longer remains in the same plane during vibration. For each main vibration, there are two components in the planes perpendicular to each other, that is, the main vibration is coupled with two transverse vibrations.

The differential equation of two-direction transverse vibration of the long cantilever structure is established by Hamilton’s principle, i.e.,

(1) $$\delta \int_{t_{1}}^{t_{2}}(T-U)\;dt+\int_{t_{1}}^{t_{2}}\delta Wdt=0$$

where T is the total kinetic energy, U is the total potential energy, δW is the virtual work done by external random aerodynamic loads, δ(·) indicates the first variation, and $t_{1}$ and $t_{2}$ are the integration time limits.

The differential equation of two-direction transverse vibration of the long cantilever system composed of an aircraft model, wind tunnel balance, and the cantilever sting is obtained as

(2) $$\begin{array}{l} \frac{\partial^{2}\left(EI_{z}\partial^{2}\upsilon /\partial x^{2}\right)}{\partial x^{2}}+\frac{\partial^{2}\left(EI_{yz}\partial^{2}w/\partial x^{2}\right)}{\partial x^{2}}+\rho A\frac{\partial^{2}\upsilon }{\partial t^{2}}=q_{y}(x,t)\\ \frac{\partial^{2}\left(EI_{y}\partial^{2}w/\partial x^{2}\right)}{\partial x^{2}}+\frac{\partial^{2}\left(EI_{yz}\partial^{2}\upsilon /\partial x^{2}\right)}{\partial x^{2}}+\rho A\frac{\partial^{2}w}{\partial t^{2}}=q_{z}(x,t) \end{array}\}$$

where $I_{z}$ and $I_{y}$ are the inertial moments of the *z*-axis and *y*-axis cross-sections respectively, $I_{yz}$ is the corresponding product of inertia, ρ is the density of long cantilever structure, and A is the cross-sectional area. The transition from Equation (1) to Equation (2) is shown in Appendix A.

### 3.2. Decoupling Design Principle of the Cantilever Sting

**Figure 3:**
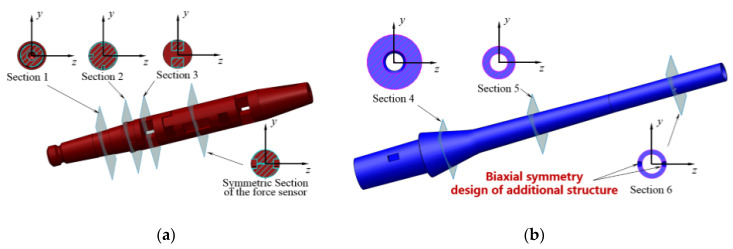
Structure characteristics of the cantilever support system. (**a**) Structure characteristics of wind tunnel balance. (**b**) Decoupling design of the cantilever sting.

The vibrations described by two vibration differential equations are mutually coupled, with the coupling terms being $\partial^{2}\left(EI_{yz}\partial^{2}w/\partial x^{2}\right)/\partial x^{2}$ and $\partial^{2}\left(EI_{yz}\partial^{2}\upsilon /\partial x^{2}\right)/\partial x^{2}$. The only way to decouple these is to make $I_{yz}=0$. As shown in Figure 3a, the wind tunnel balance section is symmetrical, regarding the *y*-axis and *z*-axis. To extend the biaxial symmetry, all the additional cantilever sting structures should be biaxially symmetrical, as shown in Figure 3b, to keep the direction of the main inertial axis of the cross-section unchanging with the *x*-axis. Therefore, a biaxial symmetry design principle is presented for the cantilever sting to keep $I_{yz}=0$.

Equation (2) can therefore be rewritten as

(3) $$\begin{array}{l} \frac{\partial^{2}\left(EI_{z}\partial^{2}\upsilon /\partial x^{2}\right)}{\partial x^{2}}+\rho A\frac{\partial^{2}\upsilon }{\partial t^{2}}=q_{y}(x,t)\\ \frac{\partial^{2}\left(EI_{y}\partial^{2}w/\partial x^{2}\right)}{\partial x^{2}}+\rho A\frac{\partial^{2}w}{\partial t^{2}}=q_{z}(x,t) \end{array}\}$$

The vibration is decoupled in the pitch plane and yaw plane, that is, the vibration characteristics in the pitch plane and yaw plane are independent of each other. Therefore, the unpredictable multidimensional vibration can be monitored by observing the vibrations in the pitch plane and yaw plane, respectively, for the long cantilever system composed of the aircraft model, wind tunnel balance, and the cantilever sting.

## 4. Unified Observation of Multidimensional Vibration

**Figure 4:**
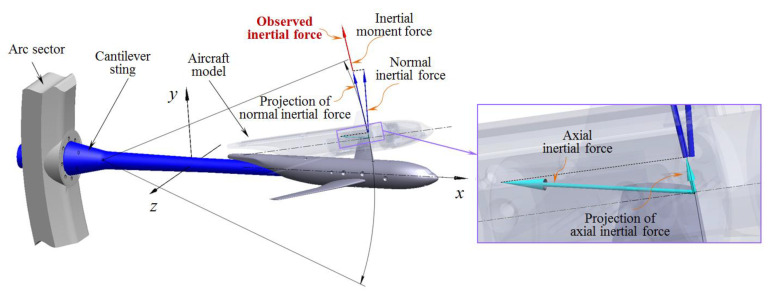
Unified observation of vibration in the pitch plane.

Based on inertial force theory, the fluctuation value of multidimensional aerodynamic force or moment is essentially the inertial force or moment of the aircraft model induced by the disturbances of complex unstable airflow. According to Section 3, unpredictable multidimensional vibration can be decoupled in the pitch plane and yaw plane. Figure 4 shows the vibration in the pitch plane.

At time t, the inertial forces are respectively generated in the *x*-axis direction, the *y*-axis direction, and the tangential direction of the pitch moment Mz. The normal inertial force can be expressed as

(4) $$F_{Iy}(t)=-m_{eq}a_{y}(t)$$

where $a_{y}(t)$ is the acceleration generated by the inertial force in the normal direction and $m_{eq}$ is the equivalent mass. $m_{eq}$ is a kind of lumped mass converted from the distributed mass of the long cantilever support system.

The axial inertial force is expressed as

(5) $$F_{Ix}(t)=-m_{eq}a_{x}(t)$$

where $a_{x}(t)$ is the acceleration generated by the inertial force in the axial direction.

The inertial moment force in pitch plane is expressed as

(6) $$F_{IMz}(t)=-m_{eq}a_{Mz}(t)$$

where $a_{Mz}(t)$ is the acceleration generated by the pitch moment Mz in the tangential direction of the pitch moment Mz.

During the vibration, the projection of the normal inertial force $F_{Iy}(t)$ in the direction of the pitch inertial moment force $F_{IMz}(t)$ can be expressed as

(7) $$F_{IyG}(t)=-m_{eq}a_{y}(t)cos\theta_{s}$$

where $\theta_{s}$ is the rotating angle at the end of the long cantilever support during the process of vibration.

The projection of the axial inertial force $F_{Ix}(t)$ in the direction of the pitch inertial moment force $F_{IMz}(t)$ can be expressed as

(8) $$F_{IyG}(t)=-m_{eq}a_{x}(t)\sin\theta_{s}$$

Within the limit vibration angle range of −90°∼90°, the normal inertial force $F_{Iy}(t)$ and the axial inertial force $F_{Ix}(t)$ exhibit projection components in the pitch inertial moment force $F_{IMz}(t)$ direction. Therefore, the resultant inertia force in the direction of the pitch inertia moment force $F_{IMz}(t)$ is defined as the observed inertia force, and the magnitude can be expressed as

(9) $$F_{IG}(t)=-m_{eq}a_{G}(t)=F_{IMz}(t)+F_{IyG}(t)+F_{IxG}(t)$$

where $a_{G}(t)$ is the observation acceleration in the direction of the pitch inertial moment force $F_{IMz}(t)$.

As the inertial force is proportional to the acceleration, the observed inertia force $F_{IG}(t)$ can be obtained from the observed acceleration $a_{G}(t)$. Thus, the unified observation of the normal inertial force $F_{IyG}(t)$, the axial inertial force $F_{IxG}(t)$, and the pitch inertial moment force $F_{IMz}(t)$ can be realized.

In the same way, the inertial force of the yaw plane can be observed uniformly. Therefore, the aircraft model’s five-dimension vibration characteristics were obtained by observing the inertial forces in the pitch and yaw planes.

## 5. Verification Experiments in the Lab and the Wind Tunnel

On the basis of dynamic theoretical analysis, the stochastic multidimensional vibration of the long cantilever support system can be uniformly observed in the pitch plane and the yaw plane, respectively. In this section, the observability of stochastic vibration in the pitch plane and the yaw plane is further verified through experimental research conducted in the lab and the wind tunnel.

### 5.1. Experimental System

**Figure 5:**
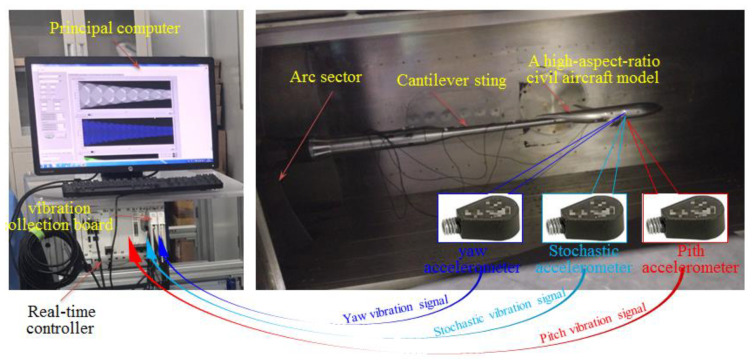
Real-time monitoring system of the stochastic multidimensional vibration.

The research object was a high-aspect-ratio civil aircraft model supported by a long cantilever support system composed of wind tunnel balance, a cantilever sting, and an arc sector. The real-time monitoring system of the stochastic multidimensional vibration is illustrated in Figure 5.

The IEPE accelerometers used in the experiment have the advantages of high precision, light weight, and small volume under the constraints of wind tunnel test conditions with narrow space, an overall complex situation, and strict aerodynamic shape requirements. The main parameters of the accelerometer are shown in Table 1, which were installed at the observation points in the center of mass of the aircraft model plane to monitor acceleration in the observation directions of the inertial forces. Acceleration was monitored in real-time using an NI real-time controller composed of a vibration collection board (NI PXI-4461 with 12-bit resolution) and a host controller (PXIe-1071DC). A principal computer was used for software operation and display of the collected data.

### 5.2. Impulse Verification Experiments in the Lab

**Figure 6:**
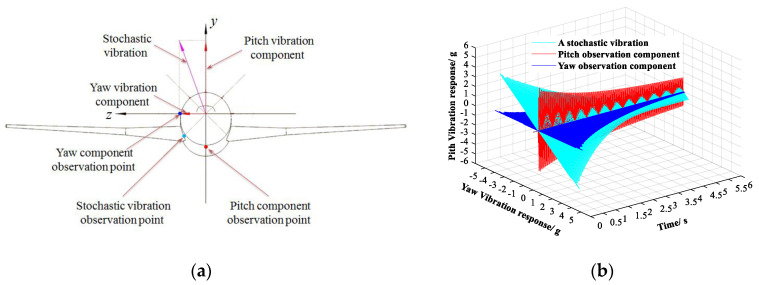
Layout of accelerometers and observation of vibration. (**a**) Layout of accelerometers. (**b**) Observation of stochastic vibration.

As shown in Figure 6a, a stochastic vibration observation point not in the pitch or yaw plane was selected, and the aircraft model’s stochastic vibration was monitored by an IEPE single-dimensional accelerometer. Two IEPE single-dimensional accelerometers were, respectively, set at the pitch component observation point and the yaw component observation point. The vibration mode frequencies included in the vibration were concentrated in the frequency range of tens to dozens of Hz, while the hammering method excited at least 0–400 Hz as a response of the system, thereby fully covering the frequency range of the system and fully reflecting the system characteristics. Hammering was used to simulate the broadband disturbances of complex unstable airflow.

The selected stochastic vibration and its vibration components in the pitch plane and yaw plane, respectively, were simultaneously monitored by the three accelerometers. These results are shown in Figure 6b.

**Figure 7:**
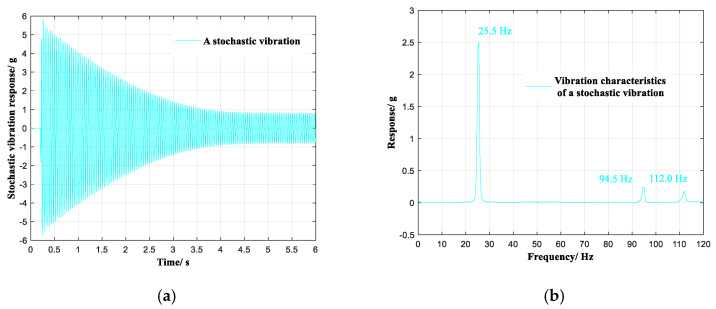
A selected stochastic vibration. (**a**) Response in the time domain. (**b**) Response in the frequency domain.

The selected stochastic vibration at the observation point is shown in Figure 7a, and analysis in the frequency domain is illustrated in Figure 7b. In the frequency region of 0~120 Hz, the selected stochastic vibration contained three vibration modes of 25.5 Hz, 94.5 Hz, and 112.0 Hz.

**Figure 8:**
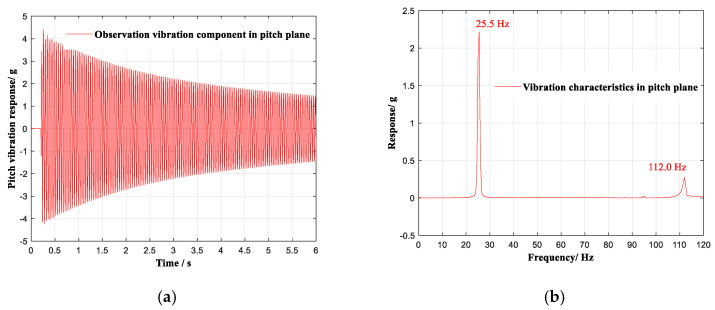
Vibration component of the selected stochastic vibration in the pitch plane. (**a**) Response in the time domain. (**b**) Response in the frequency domain.

The vibration component of the selected stochastic vibration in the pitch plane is shown in Figure 8a, and the analysis in the frequency domain is shown in Figure 8. In the frequency region of 0~120 Hz, the vibration component contained two vibration modes of 25.5 Hz and 112.0 Hz.

**Figure 9:**
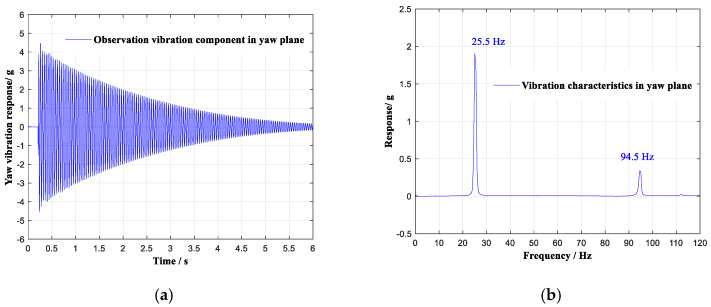
Vibration component of the selected stochastic vibration in the yaw plane. (**a**) Response in the time domain. (**b**) Response in the frequency domain.

The vibration component of the selected stochastic vibration in the yaw plane is shown in Figure 9a, and the analysis in the frequency domain is shown in Figure 9. In the frequency region of 0~120 Hz, the vibration component contained two vibration modes of 25.5 Hz and 94.5 Hz.

**Figure 10:**
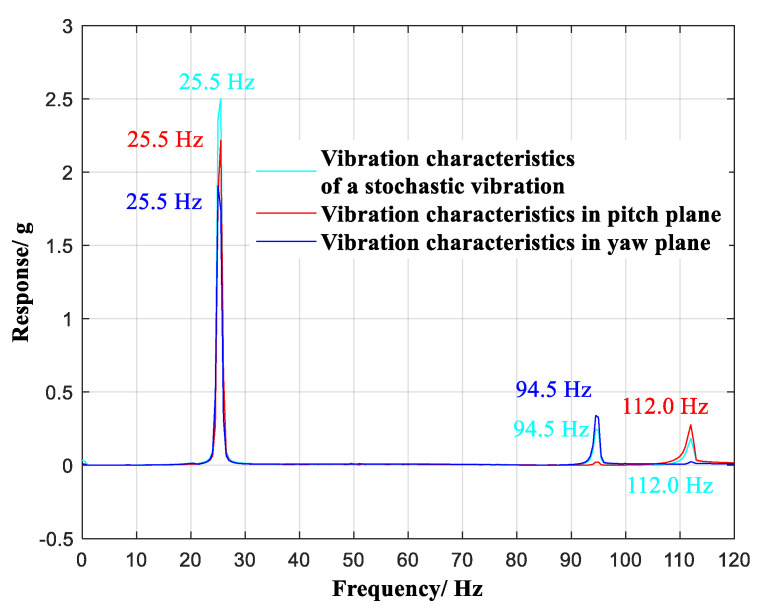
Comparison of vibration characteristics.

A comparison of the vibration characteristics of the selected stochastic vibration, i.e., the vibration component in the pitch plane and the vibration component in the yaw plane, is shown in Figure 10.

As shown in Table 2, in the frequency region of 0~120 Hz, the selected stochastic vibration contained three vibration modes of 25.5 Hz, 94.5 Hz, and 112.0 Hz. The vibration modes of 25.5 Hz and 112.0 Hz were observed via the vibration components in the pitch plane. Two vibration modes, 25.5 Hz and 94.5 Hz, were observed via the vibration components in the yaw plane. Therefore, the vibration characteristics of the selected stochastic vibration were observed to be decoupled through the vibration components in the pitch and yaw planes. Furthermore, any stochastic vibration of the wind tunnel model was observed through the vibration sensors in the pitch and yaw planes, thereby realizing the real-time monitoring of stochastic vibration while using the fewest vibration sensors.

### 5.3. Verification Experiments in Wind Tunnel

**Figure 11:**
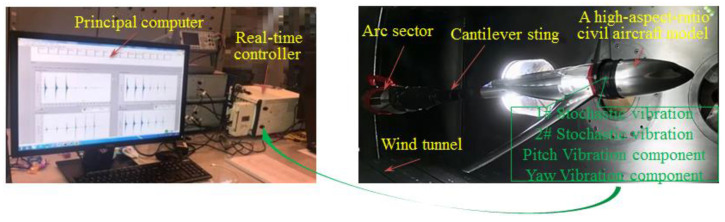
Real-time monitoring system in a continuous transonic wind tunnel.

The wind tunnel verification experiments were conducted in a continuous transonic wind tunnel. As shown in Figure 11, the high-aspect-ratio civil aircraft model was supported by long cantilever support, and installed on the attack-roll-angle adjusting mechanism.

**Figure 12:**
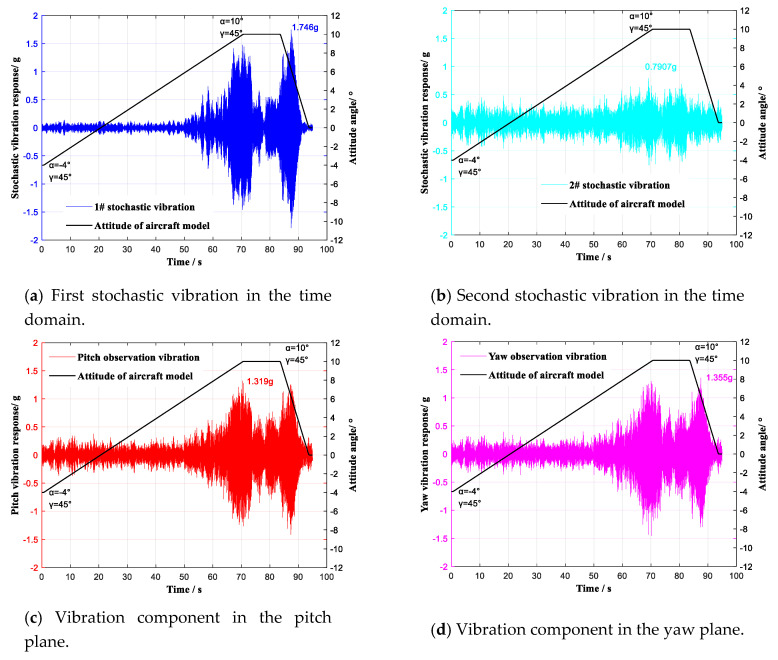
Vibration monitoring with continuous lifting (γ = 0°) of the aircraft model at Ma = 0.6.

Two stochastic vibration observation points that were not in either the pitch plane or the yaw plane were selected, and two IEPE single-dimensional accelerometers were used to monitor the aircraft model’s stochastic vibration. Two IEPE single-dimensional accelerometers were, respectively, set at the pitch component observation point and the yaw component observation point. During wind tunnel tests, the selected stochastic vibration and its vibration components in the pitch and yaw planes were simultaneously monitored by the four accelerometers. The conventional tests were performed with the attack angle α continuously ranging from −4° to 10° (roll angle γ = 45°) at Ma = 0.6 and Ma = 0.7 Ma, respectively. The observation results at Ma = 0.6 are shown in Figure 12.

**Figure 13:**
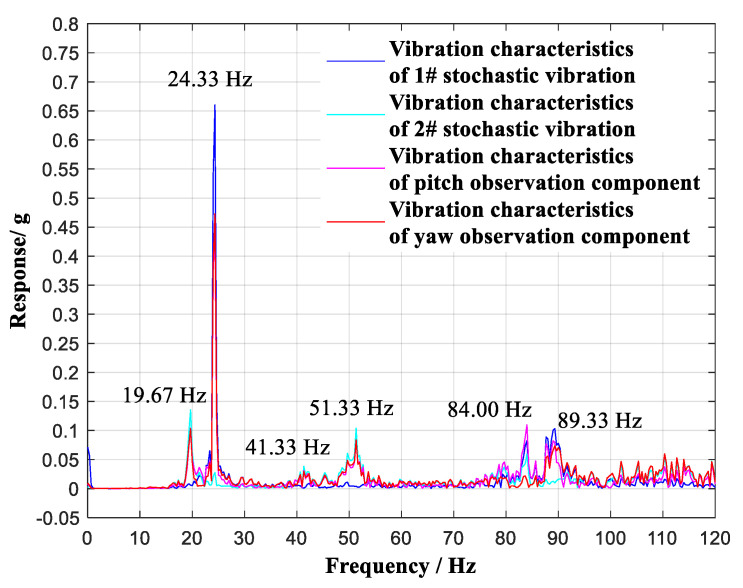
Comparison of vibration characteristics with continuous lifting (γ = 0°) of the aircraft model at Ma = 0.6.

A comparison of the vibration characteristics of the two selected stochastic vibrations, i.e., the vibration component in the pitch plane and the vibration component in the yaw plane, is shown in Figure 13.

In the frequency region of 0~120 Hz, six vibration modes of 19.67 Hz, 24.33 Hz, 41.33 Hz, 51.33 Hz, 84.00 Hz, and 89.33 Hz were identified as contrasting modes. The first stochastic vibration contained three vibration modes of 24.33 Hz, 84.00 Hz, and 89.33 Hz. The second stochastic vibration contained five vibration modes of 19.67 Hz, 24.33 Hz, 41.33 Hz, 51.33 Hz, and 84.00 Hz. Through the vibration component in the pitch plane, five vibration modes of 19.67 Hz, 24.33 Hz, 41.33 Hz, 51.33 Hz, and 89.33 Hz were observed. Through the vibration component in the yaw plane, six vibration modes of 19.67 Hz, 24.33 Hz, 41.33 Hz, 51.33 Hz, 84.00 Hz, and 89.33 Hz were observed; these are listed in Table 3. The vibration characteristics of the two selected stochastic vibrations were therefore observed to be decoupled through the vibration components in the pitch and yaw planes.

**Figure 14:**
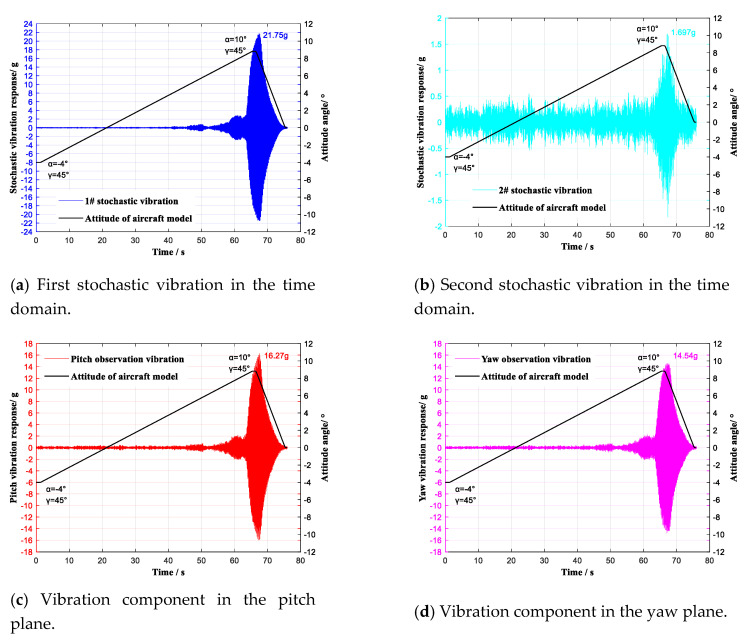
Vibration monitoring with continuous lifting (γ = 0°) of the aircraft model at 0.7 Ma.

The observation results at Ma = 0.7 are shown in Figure 14.

**Figure 15:**
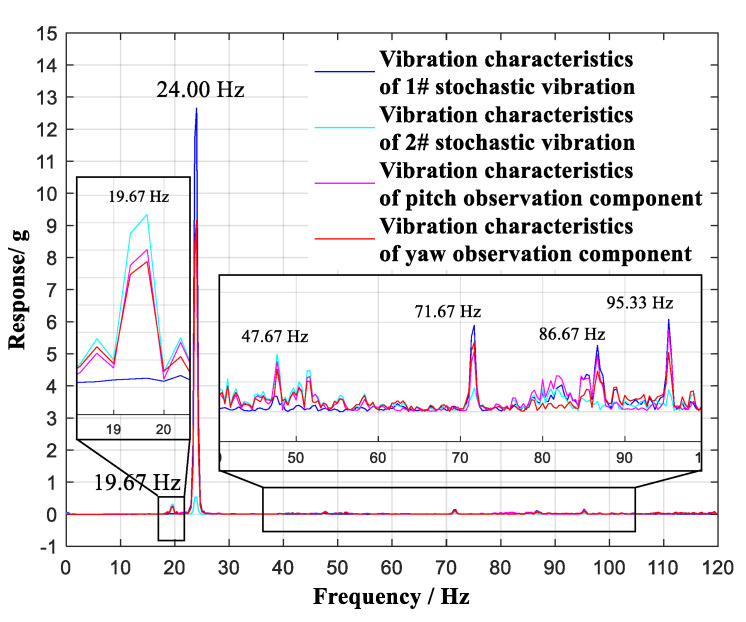
Comparison of vibration characteristics with continuous lifting (γ = 0°) of the aircraft model at Ma = 0.7.

A comparison of vibration characteristics of the two selected stochastic vibrations, i.e., the vibration component in the pitch plane and the vibration component in the yaw plane, is shown in Figure 15.

In the frequency region of 0~120 Hz, six vibration modes of 19.67 Hz, 24.00 Hz, 47.67 Hz, 71.67 Hz, 86.67 Hz, and 95.33 Hz were identified as contrasting modes. The first stochastic vibration contained five vibration modes of 24.00 Hz, 47.67 Hz, 71.67 Hz, 86.67 Hz, and 95.33 Hz. The second stochastic vibration contained five vibration modes of 19.67 Hz, 24.00 Hz, 47.67 Hz, 71.67 Hz, and 95.33 Hz. Through the vibration component in the pitch plane, six vibration modes of 19.67 Hz, 24.00 Hz, 47.67 Hz, 71.67 Hz, 86.67 Hz, and 95.33 Hz were observed. Through the vibration component in the yaw plane, six vibration modes of 19.67 Hz, 24.00 Hz, 47.67 Hz, 71.67 Hz, 86.67 Hz, and 95.33 Hz were observed; these are listed in Table 4. The vibration characteristics of the two selected stochastic vibrations were therefore observed to be decoupled through the vibration components in the pitch plane and the yaw plane.

In general, using the proposed decoupled unified observation method, the stochastic vibrations caused by disturbances in complex unstable airflow were observed to be decoupled and monitored in all dimensions via the vibration components in the pitch and yaw planes under the working conditions (α = −4~10° with γ = 45°) at Ma = 0.6 and Ma = 0.7, respectively.

## 6. Conclusions

This paper focused on the monitoring of stochastic multidimensional vibration for wind tunnel model in active vibration control, and presented a decoupling design principle for the long cantilever sting, thereby ensuring the decoupling of five-dimensional vibration in the pitch plane and the yaw plane according to the mechanical structure. A unified observation method was employed to observe the five-dimensional vibration through acceleration with inertial force theory. The stochastic five-dimensional vibration was able to be decoupled and monitored via the vibration components in the pitch plane and the yaw plane. Real-time monitoring of stochastic five-dimensional vibration was realized using minimal vibration sensors. This decoupled unified observation method provides a new idea for dimension-reduction monitoring of multidimensional vibration from structural design and sensor layouts, which perfectly serves the fields of vibration measurement and active vibration control, among others.

## Figures and Tables

**Table 1:** Main parameters of the Integral Electronic Piezoelectric (IEPE) accelerometer.

Measurement Range	Sensitivity	Size	Nonlinearity	Working Temperature	Weight
0–100 g	9.56 mV/g	3.8 × 11.36 × 6.4 mm	<±1%	−54~121°C	<3 g

**Table 2:** Comparison results observed in the lab.

	First Mode (Hz)	Second Mode (Hz)	Third Mode (Hz)
**Stochastic vibration**	25.5	94.5	112.0
**Pitch observation component**	25.5	-	112.0
**Yaw observation component**	25.5	94.5	-

**Table 3:** Comparison results at Ma = 0.6.

	1st Mode (Hz)	2nd Mode (Hz)	3rd Mode (Hz)	4th Mode (Hz)	5th Mode (Hz)	6th Mode (Hz)
**1# stochastic vibration**	-	24.33	-	-	84.00	89.33
**2# stochastic vibration**	19.67	24.33	41.33	51.33	84.00	-
**Pitch observation component**	19.67	24.33	41.33	51.33	-	89.33
**Yaw observation component**	19.67	24.33	41.33	51.33	84.00	89.33

**Table 4:** Comparison results at Ma = 0.7.

	1st Mode (Hz)	2nd Mode (Hz)	3rd Mode (Hz)	4th Mode (Hz)	5th Mode (Hz)	6th Mode (Hz)
**1# stochastic vibration**	-	24.00	47.67	71.67	86.67	95.33
**2# stochastic vibration**	19.67	24.00	47.67	71.67	-	95.33
**Pitch observation component**	19.67	24.00	47.67	71.67	86.67	95.33
**Yaw observation component**	19.67	24.00	47.67	71.67	86.67	95.33

## Appendix A

The components of displacement along the y-direction and the z-direction of each point on the elastic line can be respectively written as

(A1) υ=υ(x,t)

(A2) w=w(x,t)

Then, the displacement component along the x-direction can be expressed as

(A3) $$u_{a}=u_{a}^{\prime }+u_{a}^{\prime \prime }$$

where $u_{a}^{\prime }=-y\sin\theta$, $u_{a}^{\prime \prime }=-z\sin\varphi$, θ is the rotating angle in the y-direction, and φ is the rotating angle in the z-direction. According to the small variable theory, i.e., sinθ=θ, sinφ=φ and θ=∂υ/∂x, φ=∂w/∂x, the displacement components in three directions of each point can be rewritten as

(A4) $$u_{a}=-y\frac{\partial \upsilon }{\partial x}-z\frac{\partial w}{\partial x}$$

(A5) $$\upsilon_{a}=\upsilon$$

(A6) $$w_{a}=w$$

The kinetic energy and potential energy of the long cantilever system’s infinitesimal elements can be expressed as

(A7) $$\Delta T=\frac{\rho }{2}\left[{\left(\frac{\partial \upsilon }{\partial x}\right)}^{2}+{\left(\frac{\partial w}{\partial x}\right)}^{2}\right]△V$$

(A8) $$\Delta U=\frac{1}{2}\sigma_{x}\varepsilon_{x}\cdot \Delta V$$

(A9) $$\varepsilon_{x}=\frac{\partial u_{a}}{\partial x}=-y\frac{\partial^{2}\upsilon }{\partial x^{2}}-z\frac{\partial^{2}w}{\partial x^{2}}$$

(A10) $$\sigma_{x}=E\varepsilon_{x}$$

Then, the kinetic energy of the long cantilever system can be expressed as

(A11) $$T=\frac{1}{2}\underset{}{∭}\rho \left[{\left(\frac{\partial \upsilon }{\partial t}\right)}^{2}+{\left(\frac{\partial w}{\partial t}\right)}^{2}\right]\;dV$$

Therefore, the potential energy of the long cantilever system can be expressed as

(A12) $$U=\frac{1}{2}\underset{V}{∭}\sigma_{x}\varepsilon_{x}dV$$

By substituting Equations (A9) and (A10) into Equation (A12), the potential energy of the long cantilever system can be rewritten as

(A13) $$U=\frac{1}{2}\int_{0}^{l}\left[EI_{z}{\left(\frac{\partial^{2}\upsilon }{\partial x^{2}}\right)}^{2}+2EI_{yz}\frac{\partial^{2}\upsilon }{\partial x^{2}}\cdot \frac{\partial^{2}w}{\partial x^{2}}+EI_{y}{\left(\frac{\partial^{2}w}{\partial x^{2}}\right)}^{2}\right]dx$$

where $I_{z}$ and $I_{y}$ are the inertial moments of the *z*-axis and *y*-axis cross-sections, respectively, and $I_{yz}$ is the corresponding product of inertia.

Supposing that the distributed load on the beam are $q_{z}(x,t)$ and $q_{y}(x,t)$, and that the shear force and moment acting on the fixed support section are, $Q_{z0}$, $M_{y0}$, and, as shown in Figure 2, then the virtual work of the active force can be expressed as

(A14) $$\delta W=\int_{0}^{l}\left[q_{y}(x,t)\delta \upsilon +q_{z}(x,t)\delta w\right]\;dx+\left[Q_{y0}\delta \upsilon (0)+Q_{z0}\delta w(0)-M_{y0}\delta \frac{\partial \upsilon }{\partial x}\left(0\right)-M_{z0}\delta \frac{\partial w}{\partial x}\left(0\right)\right]$$

By substituting Equation (A11), (A13) and (A14) into Equation (1), Equation (1) can be rewritten as

(A15) $$\begin{array}{l} \delta \int_{t_{1}}^{t_{2}}\left\{\frac{1}{2}\int_{0}^{l}\rho A\left({\left(\frac{\partial \upsilon }{\partial t}\right)}^{2}+{\left(\frac{\partial w}{\partial t}\right)}^{2}\right)-\frac{1}{2}\int_{0}^{l}\left[EI_{z}{\left(\frac{\partial^{2}\upsilon }{\partial x^{2}}\right)}^{2}+2EI_{yz}\frac{\partial^{2}\upsilon }{\partial x^{2}}\cdot \frac{\partial^{2}w}{\partial x^{2}}+EI_{y}{\left(\frac{\partial^{2}w}{\partial x^{2}}\right)}^{2}\right]\right\}dxdt\\ +\int_{t_{1}}^{t_{2}}\int_{0}^{l}\left[q_{y}(x,t)\delta \upsilon +q_{z}(x,t)\delta w\right]\;dxdt+\int_{t_{1}}^{t_{2}}\left[Q_{y0}\delta \upsilon (0)+Q_{z0}\delta w(0)-M_{y0}\delta \frac{\partial \upsilon }{\partial x}\left(0\right)-M_{z0}\delta \frac{\partial w}{\partial x}\left(0\right)\right]dt=0 \end{array}$$

As the instantaneous motion at $t_{1}$ to $t_{2}$ is given, the first term of kinetic energy variation in Equation (A15) is expressed as

(A16) $$\delta \int_{t_{1}}^{t_{2}}\frac{1}{2}\int_{0}^{l}\rho A{\left(\frac{\partial \upsilon }{\partial t}\right)}^{2}dxdt=\int_{t_{1}}^{t_{2}}\int_{0}^{l}\rho A\frac{\partial^{2}\upsilon }{\partial t^{2}}\delta \upsilon dxdt$$

In the same way, the remaining term of kinetic energy variation in Equation (A15) can be obtained by

(A17) $$\delta \int_{t_{1}}^{t_{2}}\frac{1}{2}\int_{0}^{l}\rho A{\left(\frac{\partial w}{\partial t}\right)}^{2}dxdt=-\int_{t_{1}}^{t_{2}}\int_{0}^{l}\rho A\frac{\partial^{2}w}{\partial t^{2}}\delta wdxdt$$

The first term of potential energy variation in Equation (A15) is expressed as

(A18) $$\begin{array}{l} \delta \int_{t_{1}}^{t_{2}}\frac{1}{2}\int_{0}^{l}EI_{z}{\left(\frac{\partial^{2}\upsilon }{\partial x^{2}}\right)}^{2}dxdt=\int_{t_{1}}^{t_{2}}\int_{0}^{l}EI_{z}\frac{\partial^{2}\upsilon }{\partial x^{2}}\delta \frac{\partial^{2}\upsilon }{\partial x^{2}}dxdt\\ =\int_{t_{1}}^{t_{2}}EI_{z}\frac{\partial^{2}\upsilon }{\partial x^{2}}\delta \frac{\partial \upsilon }{\partial x}|\overset{l}{\underset{0}{}}dt-\int_{t_{1}}^{t_{2}}\frac{\partial \left(EI_{z}\frac{\partial^{2}\upsilon }{\partial x^{2}}\right)}{\partial x}\delta \upsilon |\overset{l}{\underset{0}{}}dt+\int_{t_{1}}^{t_{2}}\int_{0}^{l}\frac{\partial^{2}\left(EI_{z}\frac{\partial^{2}\upsilon }{\partial x^{2}}\right)}{\partial x^{2}}\delta \upsilon dxdt \end{array}$$

In the same way, the remaining two terms of potential energy variation in Equation (A15) can be obtained by

(A19) $$\begin{array}{l} \delta \int_{t_{1}}^{t_{2}}\int_{0}^{l}EI_{yz}\frac{\partial^{2}\upsilon }{\partial x^{2}}\cdot \frac{\partial^{2}w}{\partial x^{2}}dxdt=\int_{t_{1}}^{t_{2}}\int_{0}^{l}\left(EI_{yz}\frac{\partial^{2}\upsilon }{\partial x^{2}}\delta \frac{\partial^{2}w}{\partial x^{2}}+EI_{yz}\frac{\partial^{2}w}{\partial x^{2}}\delta \frac{\partial^{2}\upsilon }{\partial x^{2}}\right)dxdt\\ =\int_{t_{1}}^{t_{2}}EI_{yz}\frac{\partial^{2}\upsilon }{\partial x^{2}}\delta \frac{\partial w}{\partial x}|\overset{l}{\underset{0}{}}dt-\int_{t_{1}}^{t_{2}}\frac{\partial \left(EI_{yz}\frac{\partial^{2}\upsilon }{\partial x^{2}}\right)}{\partial x}\delta w|\overset{l}{\underset{0}{}}dt+\int_{t_{1}}^{t_{2}}\int_{0}^{l}\frac{\partial^{2}\left(EI_{z}\frac{\partial^{2}\upsilon }{\partial x^{2}}\right)}{\partial x^{2}}\delta wdxdt\\ +\int_{t_{1}}^{t_{2}}EI_{yz}\frac{\partial^{2}w}{\partial x^{2}}\delta \frac{\partial \upsilon }{\partial x}|\overset{l}{\underset{0}{}}dt-\int_{t_{1}}^{t_{2}}\frac{\partial \left(EI_{yz}\frac{\partial^{2}w}{\partial x^{2}}\right)}{\partial x}\delta \upsilon |\overset{l}{\underset{0}{}}dt+\int_{t_{1}}^{t_{2}}\int_{0}^{l}\frac{\partial^{2}\left(EI_{yz}\frac{\partial^{2}w}{\partial x^{2}}\right)}{\partial x^{2}}\delta \upsilon dxdt \end{array}$$

(A20) $$\begin{array}{l} \delta \int_{t_{1}}^{t_{2}}\frac{1}{2}\int_{0}^{l}EI_{y}{\left(\frac{\partial^{2}w}{\partial x^{2}}\right)}^{2}dxdt=\int_{t_{1}}^{t_{2}}\int_{0}^{l}EI_{y}\frac{\partial^{2}w}{\partial x^{2}}\delta \frac{\partial^{2}w}{\partial x^{2}}dxdt\\ =\int_{t_{1}}^{t_{2}}EI_{y}\frac{\partial^{2}w}{\partial x^{2}}\delta \frac{\partial w}{\partial x}|\overset{l}{\underset{0}{}}dt-\int_{t_{1}}^{t_{2}}\frac{\partial \left(EI_{y}\frac{\partial^{2}w}{\partial x^{2}}\right)}{\partial x}\delta w|\overset{l}{\underset{0}{}}dt+\int_{t_{1}}^{t_{2}}\int_{0}^{l}\frac{\partial^{2}\left(EI_{y}\frac{\partial^{2}w}{\partial x^{2}}\right)}{\partial x^{2}}\delta wdxdt \end{array}$$

As the instantaneous motion at $t_{1}$ to $t_{2}$ is given, the variations δυ(0), $\delta \frac{\partial \upsilon }{\partial x}\left(0\right)$, δw(0), and $\delta \frac{\partial w}{\partial x}\left(0\right)$ on the boundary should be 0 for the displacement boundary conditions. The force boundary condition is arbitrary, as are δυ and δw in the domain. Equation (A15) can therefore be expressed as

(A21) $$\begin{array}{l} \int_{0}^{l}\left[\frac{\partial^{2}\left(EI_{z}\partial^{2}\upsilon /\partial x^{2}\right)}{\partial x^{2}}+\frac{\partial^{2}\left(EI_{yz}\partial^{2}w/\partial x^{2}\right)}{\partial x^{2}}+\rho A\frac{\partial^{2}\upsilon }{\partial t^{2}}-q_{y}(x,t)\right]\delta \upsilon dt=0\\ \int_{0}^{l}\left[\frac{\partial^{2}\left(EI_{y}\partial^{2}w/\partial x^{2}\right)}{\partial x^{2}}+\frac{\partial^{2}\left(EI_{yz}\partial^{2}\upsilon /\partial x^{2}\right)}{\partial x^{2}}+\rho A\frac{\partial^{2}w}{\partial t^{2}}-q_{z}(x,t)\right]\delta wdt=0 \end{array}\}$$
